# Epicardial unipolar radiofrequency ablation for left ventricular aneurysm related ventricular arrhythmia

**DOI:** 10.1186/1749-8090-8-124

**Published:** 2013-05-07

**Authors:** Yang Yu, Ming-xin Gao, Cheng-xiong Gu

**Affiliations:** 1 Department of Cardiac Surgery Beijing An Zhen Hospital, Capital Medical University, Beijing, 100029, China

**Keywords:** Radiofrequency ablation, Left ventricular aneurysm, Ventricular tachycardia, Off-pump coronary artery bypass

## Abstract

We report a case of a 62-year-old Chinese man with typical triple-vessel lesions and apical left ventricular aneurysm accompanied with ventricular tachycardia. Off-pump coronary artery bypass (OPCAB) grafting was performed in combination with epicardial unipolar radiofrequency ablation and linear closure of left ventricular aneurysm. The patient recovered well without postoperative complications. Holter monitoring showed no recurrence of the ventricular arrhythmia and the attack frequency of arrhythmia decreased significantly. The patient has been angina-free for 25 months since the operation and shows increasing exercise tolerance. Thus, left ventricular aneurysm plication combined with epicardial unipolar radiofrequency ablation during OPCAB may be beneficial for patients with ventricular aneurysm and preoperative malignant ventricular arrhythmia.

## Background

Left ventricular aneurysm (LVA) is one of the serious complications following acute myocardial infarction. Surgery is considered as an effective treatment for this disorder. However, it has been reported that the incidence rate of late sudden death caused by malignant ventricular arrhythmia (VA) after LVA repair can reach 36.8% of the total deaths [1]. Thus, intra and postoperative anti-arrhythmic therapy is advisable in patients undergoing LVA repair [1]. Radiofrequency ablation is a proven effective procedure for treating VA [2]. Here, we reported one case of patient with ventricular tachycardia undergoing LVA repair plus epicardial unipolar radiofrequency ablation, and we found that the combined procedure showed promising efficacy and patient outcomes.

## Case presentation

A 62-year-old Chinese man who had myocardial infarction on anterior wall was recommended for cardiac surgery after diagnosed with unstable angina. His cardiac function was grade III according to the New York Heart Association classification. Coronary angiography showed triple-vessel lesions. Echocardiography revealed paradoxical cardiac movements in the apex of the left ventricle in the systolic phase without mural thrombosis. The left ventricular ejection fraction was 0.40 and the left ventricular end-diastolic diameter was 55 mm. Preoperative 12-lead electrocardiography (ECG) showed premature ventricular contractions (PVC). 12-lead Holter monitoring confirmed the presence of frequent multifocal VA (26400/24 h) and paroxysmal ventricular tachycardia. The patient had non-sustained ventricular tachycardia, and did not show mural thrombi. The patient took at least three kinds of anti-arrhythmic drugs including beta-blocker, lidocaine, and amiodarone to reduce the occurrence of VA before the operation. However, those anti-arrhythmic drugs did not seem effective for the patient.

Standard median sternotomy incision was used to expose the heart. Off-pump coronary artery bypass (OPCAB) grafting was performed. A left internal mammary artery was grafted to the left anterior descending artery and a saphenous vein was anastomosed to the diagonal branch, the obtuse marginal artery, and the right coronary post-descending artery sequentially. The border of the aneurysm wall was clear on the beating heart.

**Figure 1:**
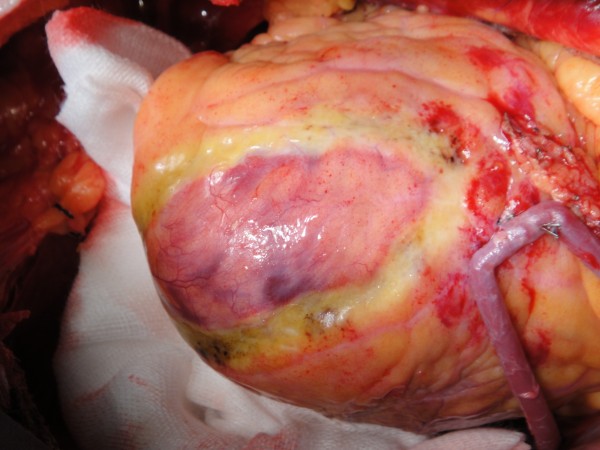
A circular epicardial ablation line was made along the border between the aneurysm wall and the surrounding normal myocardial tissue using the unipolar ablation pen.

A bipolar intramural electrode placed at the base of right ventricle via right femoral vein was used to record a reference electrogram. Ventricular tachycardia appeared spontaneously during the operation. We used more than 40 predetermined locations on the left ventricle to record bipolar potential to construct ventricular epicardial depolarization map. The time point when the epicardial spike moved the most rapidly, approximately 7 ms, represented the moment at which electrical force passed between the bipolar references. The locations where the rapid spike movement occurred were marked. Connecting these locations resulted in a circular area along the border between the aneurysm wall and the surrounding normal myocardial tissue. The circular area was the target for radiofrequency ablation. Other locations showed a spiking time longer than 7 ms. Unipolar radiofrequency ablation was performed by using a Medtronic system, which consisted of a Cardioblate 68000 ablation host and a Cardioblate unipolar pen. The ablation energy was set at 30w without temperature control, and the duration of radiofrequency discharge was set at 20–30s. We first performed a circular epicardial ablation along the borderline using the unipolar ablation pen (Figure 1).

**Figure 2:**
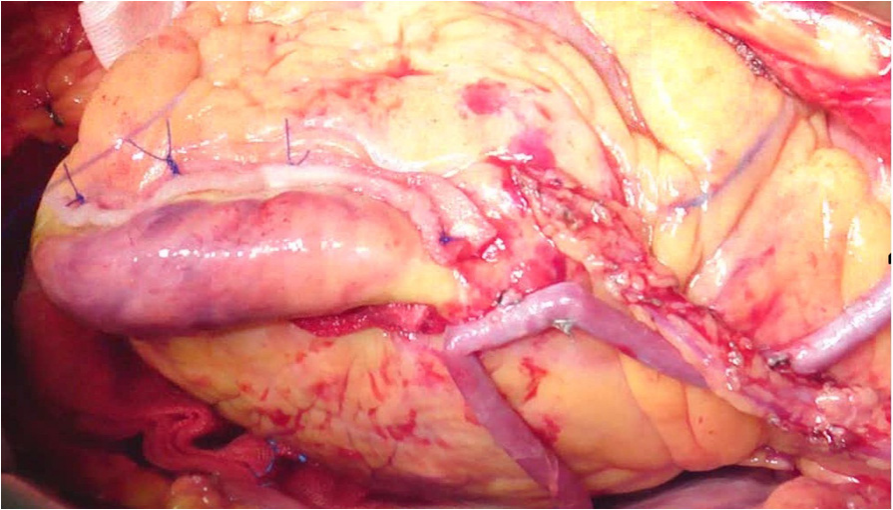
LVA was repaired by linear closure using interrupted mattress stitches with Surgipro-843 sutures and two strips of felt for reinforcement.

**Figure 3:**
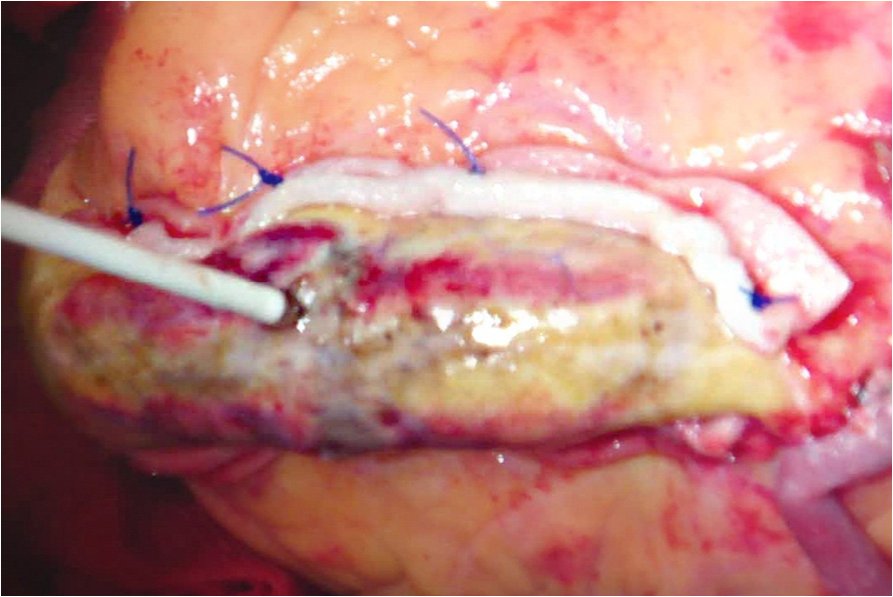
A cross-shaped ablation line was made on the epicardium at the center of LVA.

The left ventricular aneurysm was then repaired by linear closure [3]. The edges were readapted with two strips of felt for reinforcement, parallel to the long axis of the left ventricle. Interrupted mattress stitches were used with Surgipro-843 sutures (Figure 2). Sutures were placed further apart on the tissue edges rather than on the felt strips. This strategy improved the restoration of the normal shape of ventricle. Finally, we performed cross-shaped ablation on the epicardium at the central zone of the aneurysm (Figure 3).

In this case, we found that radiofrequency ablation significantly reduced the frequency of accidental premature ventricular contraction to less than one per minute, and the same type of preoperative arrhythmia did not occur within 30 min after the operation. In addition, we compared the effect of overdrive pacing on the patient’s cardiac rhythm before and after radiofrequency ablation. We found that before the ablation, overdrive pacing accelerated the patient’s cardiac rhythm; while after the ablation, overdrive pacing did not have any effect on the patient’s cardiac rhythm. This observation indicates that the radiofrequency ablation procedure could affect the reentrant mechanism underlying the VA in this patient. VA did not occur when sinus rhythm reached 120–130 beats per min responding to intravenous perfusion of isoprenaline [4]. Incision was carefully washed before the chest was closed. An accurate assessment of the effectiveness of radiofrequency ablation would be a re-induction of reentry via burst pacing or programmed stimulation after the operation. We did not perform this test in this case because the patient had spontaneous ventricular arrhythmia. We will include this test in our future investigation.

The patient recovered well without postoperative complications and was discharged at day 7 after the operation. He has not taken any anti-arrhythmic medicine since the operation. Holter monitoring at the seventh day after the operation showed no recurrence of the same VA and the amount of arrhythmia decreased to 120/24 h. The patient came back to follow-up visit six months after the operation and had no complaints for heart symptoms. His cardiac function was improved to grade II. Holter monitoring at the six-month follow-up visit showed occasional arrhythmia. We did not perform additional Holter monitoring test on the patient beyond six-month follow-up, because we think that the test might provide limited information regarding the correlation between the incidence of VA and LVA repair, although the test result reflects how well the VA condition has been improved by the operation. Currently, we are still exploring new electrophysiological approaches that can accurately identify the association between the incidence of VA and the location of aneurysm. In addition to the electrophysiological test, we performed other examination to assess the overall postoperative outcome of the patient. Echocardiography revealed ejection fraction of 0.50 and left ventricular end-diastolic diameter of 50 mm at the six-month follow-up visit. The patient has been angina-free for 25 months since the operation and showed increasing exercise tolerance.

## Discussion

The incidence rate of LVA is10–38% among patients with myocardial infarction [5]. The 5- and 10-year survival rate of patients with LVA is 58–80% and 34%, respectively. The common therapies for this condition include linear closure and endoventricular patch plasty, both of which are performed during cardiopulmonary bypass. In recent years, ventricular aneurysm repair in OPCAB has been shown to be effective and feasible as well [3]. Although aneurysm plasty can effectively improve cardiac function and patient’s spontaneous symptoms, long-term follow-up has revealed high rate of sudden death caused by malignant arrhythmia after LVA repair due to the fact that aneurysm frequently causes anatomical and electrophysiological changes in the tissue that can lead to malignant VA [6]. Recent studies have also shown that VA is primarily caused by reentrant cycle, and the associated tissue structures of VA are surviving myocardial islands, necrotic tissue, and fibrous tissues located near the border between infarcted and normal myocardium [7]. These tissues increase local autorhythmicity, which results in VA in the presence of a reentrant pathway.

Anti-arrhythmic drugs, which can inhibit autorhythmicity and prolong refractory period of myocardial cells, are beneficial for patients with increased focal myocardial automaticity but not effective for this patient who had reentrant VA caused by ventricular aneurysm. Implantable cardiac defibrillator (ICD) implantation is an effective therapeutic approach to decrease death risk caused by malignant arrhythmia. However, the patient did not choose ICD implantation because ICD cannot eliminate the original point or reentrant pathways of VA and would cause great physical and psychological stress to the patient [8]. In addition, the relatively high price of ICD was another reason for restricting its use in this case. Thus, we performed epicardial unipolar radiofrequency ablation combined with linear closure of LVA to treat ventricular aneurysm related VA for this patient. Although the combined procedure was effective for this particular patient, it should not be considered as a general replacement for ICD implantation, which remains an effective therapeutic approach for recurrent VA.

In addition to ventricular aneurysm excision, some physicians have used encircling endocardial ventriculotomy, subtotal endocardial scar resection, or encircling endocardial cryoablation to treat patients with ventricular aneurysms and arrhythmias. Myocardial incision and cardiopulmonary bypass may cause additional tissue damages, which may impair cardiac function and increase surgical risks. In this case, the epicardial mapping showed that the key elements such as the reentrant cycle were located in the epicardial layer. Therefore, simple endocardial treatment may not effectively block the reentrant cycle. Recently, radiofrequency catheter ablation techniques for treating post-infarction VA have been greatly advanced [9]. These techniques include three-dimensional mapping and ablation of VAs induced by post-infarction hemodynamic instability, closed-chest endocardial mapping and ablation, and saline-perfused radiofrequency ablation [9]. The addition of radiofrequency ablation to surgical therapy for ventricular aneurysm may improve therapeutic efficacy.

We performed circular radiofrequency ablation along the borderline between ventricular aneurysm and normal myocardium using a unipolar radiofrequency ablation pen. We also conducted cross-shaped ablation in the center of ventricular aneurysm. Our result showed that these two procedures effectively destroyed or isolated reentrant cycles in the periphery or the center of ventricular aneurysm, thereby reducing or eliminating VAs. Intra- and postoperative tests showed that malignant arrhythmias could be effectively controlled by these procedures.

We performed epicardial activation sequence mapping on the patient during the operation. Although we mapped the reentry circuit, we did not search for the critical isthmus. It is likely that the critical isthmus for the arrhythmia was contained in the scar tissue since the surgical procedure resulted in an excellent postoperative outcome for this patient. We are currently exploring novel electrophysiological approaches that allow us to efficiently perform activation sequence mapping of circuit reentry and to identify critical isthmuses by pacing criteria during VT.

We think that linear closure of LVA might have the following advantages: to restore the normal shape of heart, reduce intraoperative blood loss, and increase patient’s cardiac ejection fraction and graft blood flow, which then alleviate cardiac ischemia and reduce the incidence of arrhythmia. In this case, simultaneous off-pump CABG and linear closure of aneurysm might further reduce postoperative complication and the risk of arrhythmia that is associated with surgical scar caused by aneurysm resection. It has been proposed that LVA repair may reduce the incidence of arrhythmia by reducing left ventricular size because myocardial stretching is thought to contribute to the development of arrhythmia [1]. However, study by Bartels et al. shows that high incidence of late sudden death is significantly associated with postoperative ventricular tachyarrhythmia in patients who have undergone LVA repair without concomitant anti-arrhythmic surgery [1]. In this study, we treated the aneurysm related VA with both surgical and electrophysiological approaches and performed a concomitant radiofrequency ablation combined with OPCAB and LVA repair on the patient. The patient’s postoperative outcome is good. Nevertheless, a comprehensive investigation to compare the efficacy of linear closure of aneurysm alone with that of a concomitant anti-arrhythmic intervention is required to provide strong evidence to support the efficacy of concomitant ablation procedure.

Although the postoperative outcome of simultaneous OPCAB, linear closure of aneurysm, and radiofrequency ablation is good for this particular patient, some limitations should be considered to interpret the observation and to use this procedure in future. First, due to patient’s spontaneous VA, we did not perform programmed electrical stimulation to compare prior and postoperative status of VA. Thus, the general efficacy of the combined procedure still needs further investigation. Second, although we used 12-lead Holter monitoring test to verify the reduction of arrhythmia after the operation, we think that Holter monitoring might not be able to identify the correlation between the incidence of VA and the location of aneurysm. We are currently exploring new electrophysiological approaches to address this issue. Third, ICD is an effective therapeutic method for recurrent and life-threatening VA, but it might not remove the cause for aneurysm related VA. In addition, ICD is not only expensive, but also might cause physical and psychological side effects for patients, such as discomfort at the ICD site, anxiety, and depression. Thus, we think that ICD implantation might not be a preferred strategy for patients suffering from LVA related VA. We did not use ICD implantation for this particular patient. However, if the patient developed recurrent frequent premature ventricular contraction or ventricular tachycardia or ventricular fibrillation after the operation, ICD would be the best option. Moreover, we still need to further investigate whether the combined surgical procedure or each individual procedure could reduce the incidence of future cardiomyopathy substantially. To further confirm the efficacy of the procedure of combining ablation with aneurysm closure and OPCAB, we are currently conducting a retrospective study to compare the long-term outcome of OPCAB plus aneurysm closure with that of OPCAB plus aneurysm and radiofrequency ablation.

## Conclusions

In this case, we performed left ventricular aneurysm plication plus epicardial radiofrequency ablation therapy during OPCAB. We not only corrected the structural and functional abnormalities associated with aneurysm, but also provided electrophysiological treatment and avoided the risk associated with cardiopulmonary bypass in this case. Thus, this operation may be beneficial for patients with ventricular aneurysm and preoperative malignant arrhythmia. The long-term efficacy of this procedure needs to be further investigated.

## Consent

Written informed consent was obtained from the patient for publication of this Case report and any accompanying images. A copy of the written consent is available for review by the Editor-in-Chief of this journal.

## Abbreviations

OPCAB: Off-pump coronary artery bypass; LVA: Left ventricular aneurysm; VA: Ventricular arrhythmia; ECG: Electrocardiography; PVC: Premature ventricular contraction; ICD: Implantable cardiac defibrillator.

## Competing interests

We declared that we have no competing interests.

## Authors’ contributions

YY drafted the manuscript. MG collected the clinical material and performed the statistical analysis. CG proposed the study and helped to draft the manuscript. All authors read and approved the final manuscript.
